# Differences in the Structure and Protein Expression of Femoral Nerve Branches in Rats

**DOI:** 10.3389/fnana.2020.00016

**Published:** 2020-04-08

**Authors:** Shuai Wei, Qian Hu, Xiaoqing Cheng, Jianxiong Ma, Xuezhen Liang, Jiang Peng, Wenjing Xu, Xun Sun, Gonghai Han, Xinlong Ma, Yu Wang

**Affiliations:** ^1^Tianjin Hospital Tianjin University, Tianjin, China; ^2^Institute of Orthopedics, Chinese People’s Liberation Army (PLA) General Hospital, Beijing, China; ^3^Co-Innovation Center of Neuroregeneration, Nantong University, Nantong, China; ^4^Department of Geriatrics, The Second People’s Hospital of Nantong, Nantong, China; ^5^The First Clinical Medical School, Shandong University of Traditional Chinese Medicine, Shandong, China; ^6^The First People’s Hospital of Yunnan Province, Kunming, China

**Keywords:** cutaneous branch, femoral nerve, muscular branch, protein expression, G-ratio, ultrastructure

## Abstract

Treatment for peripheral nerve injury remains limited. The inherent differences between motor and sensory nerve fibers in peripheral nerves should be considered to improve the effects of clinical treatment of peripheral nerve injury. In this study, we investigated the differences in protein expression and ultrastructure between the cutaneous and muscular branches of the femoral nerve in rats. Our results suggest that the cutaneous branch of the femoral nerve mainly contains sensory nerve fibers and few motor nerve fibers; Correspondingly, many motor nerve fibers and few sensory nerve fibers were observed in the muscular branch of the femoral nerve, which indicate that two branches of femoral nerve are mixed nerve. The mean thickness of the myelin sheath and basement membrane of the medullated fibers in the muscular branch of the femoral nerve was greater than that of the cutaneous branch of the femoral nerve. However, the cutaneous branch has a larger G-ratio. Gene Ontology enrichment analysis revealed that the cellular component term extracellular space was the most highly enriched, and more genes were upregulated in the muscular branch of the femoral nerve. Meanwhile, the expression of key proteins were validated by Western Blot, and immunofluorescence targets the expression of key proteins, which is consistent with the enrichment analysis of Gene Ontology. In conclusion, inherent differences in protein expression and ultrastructure were observed between the cutaneous and muscular branches of the femoral nerve in rats, which should be considered in future studies on the treatment of peripheral nerve injuries.

## Significance

With the development of tissue engineering and 3D printing, it is a new trend to treat peripheral nerve defects with tissue engineering nerve. To prepare tissue nerve grafts that can more accurately imitate the structure and function of natural nerves for peripheral nerve defect, we must have a deeper understanding of different kinds of peripheral nerves. In this study, we investigated the differences in protein expression and ultrastructure between the cutaneous and muscular branches of the femoral nerve in rats. This finding has a high potential for the preparation of different kinds of tissue engineering nerve grafts.

## Introduction

Peripheral nerve injury often leads to limb dysfunction. Although peripheral nerve injuries have been studied for decades, their treatment remains a challenge (Sameem et al., 2011). More specifically, there is an inherent difference between sensory and motor nerve fascicles in the peripheral nervous system (PNS; Turkof et al., 2006). Axon regeneration and remyelination underlie peripheral nerve functional recovery following injury (Chen et al., 2007). Early studies have demonstrated that sensory and motor pathways might differ, particularly that motor axons escape from cutaneous pathways to reinnervate the appropriate motor pathway (Langley and Anderson, 1904). There are inherent differences between motor and sensory nerve fibers among peripheral nerves, and studies have revealed that the differences between motor and sensory nerves play an important role in nerve fiber regeneration during Wallerian degeneration (Abdullah et al., 2013).

Autologous nerve transplantation remains the gold standard of treatment in the absence of tension-reducing sutures for peripheral nerve injuries with a nerve gap defined as the distance between two ends of a severed nerve, which results from nerve retraction and/or loss of tissue from injury (Sachanandani et al., 2014). However, tissue-engineered nerves and acellular nerve grafts (ANGs) have shown promise for use in long-distance nerve injuries. ANGs have achieved good results in primate experiments (Wang et al., 2010) and Avance^®^ (AxoGen Inc., Alachua, FL, USA) is a commercially available acellular allogeneic nerve graft product that has been applied in clinical case studies (Karabekmez et al., 2009). However, ANGs with complete immunogenicity has not been produced (Zhang et al., 2010). Therefore, some researchers have turned their interest to synthetic materials for use as neural scaffolds. The tissue-engineered nerve grafts used to repair nerve injury are mainly hollow ducts and do not accurately replicate the three-dimensional (3D) structure of the natural nerve or the proportion of each component. Three-dimensional printing technology has been successfully used in many fields, such as vascular (Lee et al., 2016), bone repair material (He et al., 2015), and cartilage repair material (Cui et al., 2012). Tissue-engineered nerve grafts with 3D printing technology will promote further development of peripheral injury repair materials if they can overcome the deficiencies of the hollow nerve conduit and can replicate the natural nerve in structure and composition. However, it remains unknown whether there is a difference in the ultrastructure between sensory and motor nerves.

Many animal models are used to study peripheral nerve injury. The most commonly used nerves are the sciatic and femoral nerves of rats (Cobianchi et al., 2014; Karegar and Mohammadi, 2015). Because of the high purity of the muscular and saphenous nerve branches of the femoral nerve, they are often used to study chemotactic regeneration of peripheral nerves (Cai et al., 2017). Understanding the inherent molecular differences between sensory and motor nerves is important to improve the functional outcome of peripheral nerve regeneration and promote further development of tissue-engineered nerve grafts with 3D printing technology. In this study, we investigated differentially expressed proteins and the ultrastructure of the cutaneous and muscular branches of the femoral nerve in rats.

## Materials and Methods

### Sprague–Dawley Rats and Tissue Collection

The experimental operations related to experimental animals were approved by the Ethics Committee of the Chinese PLA General Hospital, China. Meanwhile, all procedures correlated to experimental animals were performed following the Guides for the Care and Use of Laboratory Animals. Thirty 8-week-old male adult Sprague–Dawley (SD) rats, weight 200–250 g, were anesthetized by intraperitoneal injection of moderate sodium pentobarbital (30 mg/ml, 30 mg/kg) for tissue collection, and an overdose of sodium pentobarbital for euthanasia (30 mg/ml, 0.6 ml). Thirty rats were numbered, 10 of them were used for histological and structural evaluation and twenty rats were used in the protein microarray analysis and Western Blot by using the computer-generated random number table. It must be noted that the rats used for transmission electron microscopy (TEM) should be perfusion fixed before obtaining nerve. A 2 cm long oblique incision was made along the groin, and the muscle tissue along the iliac psoas muscle was bluntly separated. Afterwards, the femoral nerve with a shape of Y can be seen. The trunk and branches of the femoral nerve were exposed from both sides, and the femoral nerve trunk was isolated with micro forceps 1 cm from the bifurcation of the femoral nerve and cut in the proximal end. The cutaneous branch of the femoral nerve companioned with great saphenous vein should be isolated carefully and cut by the same method, where there is a lot of fat tissue. At last, the muscular branch of the femoral nerve was cut into the muscles. Sterile micro forceps were used during the procedure to carefully hold the broken end of the nerve, so as not to cause a traction injury to the nerve tissue.

### Specimen Processing

The bifurcation of the femoral nerve was removed off after obtaining the nerve trunk, cutaneous branch, and muscular branch of the femoral nerve. The sample was stored at −80°C for the protein microarray analysis and Western Blot, and the remainder was processed for histological and ultrastructural evaluation.

The protein samples were extracted from the cutaneous and muscular branches of the femoral nerve of the rats and lysed in radioimmunoprecipitation lysis buffer containing a protease inhibitor cocktail (Pulilai, Beijing, China). Protein concentration was determined with the Pierce BCA Protein Assay Kit (Thermo Fisher Scientific, Waltham, MA, USA). A microarray analysis was performed using the Rat Cytokine Array 67 (RayBiotech, Norcross, GA, USA). Signals were visualized using the InnoScan 300 microarray scanner (Innopsys, Carbonne, France) in the Cy3 dye wavelength range (green channel). The samples were analyzed using GSR-CAA-67-SW (RayBiotech Life), which is a Q-Analyzer tool specific for this array.

The nerve trunk and cutaneous branches of the femoral nerve were immediately embedded in original collagen tube (OCT) medium and frozen at −20°C for sectioning. The nerve segments of the cutaneous and muscular branches were rapidly fixed in precooled 2.5% glutaraldehyde for 2 h for transverse ultrathin sectioning, and post-fixed in 1% osmium tetroxide solution for 2 h at 4°C.

### Analysis of Differentially Expressed Proteins

The expression levels of proteins at cutaneous branches of the femoral nerve were compared with the muscular branches. Proteins with an expression fold change >2 or < −2 and adjusted *p*-value < 0.05 were considered significantly differentially expressed.

### Bioinformatics Analysis

#### Protein-Protein Interaction (PPI) Network Construction and Analysis

The GeneMANIA database (Zuberi et al., 2013) is a gene and protein analysis tool designed to predict PPIs. Various proteins were mapped using GeneMANIA to evaluate the relationships among them. Then, the PPI networks were constructed using Cytoscape software^1^. The results were obtained by entering target genes from the previous step into the search column.

#### GO and KEGG Analyses

The DAVID database provides a comprehensive set of functional annotation, visualization, and integrated discovery tools to allow researchers to understand the biological implications of a large list of genes (Huang da et al., 2009). The database provides typical batch annotation and GO terminology-rich and KEGG pathway analyses to highlight the most relevant KEGG pathways and GO terms associated with a given gene list. GO analysis consists of three categories: biological processes (BP), molecular functions (MF), and cell components (CC). We used a cutoff *P*-value ≤ 0.05 (corrected using the Benjamini–Hochberg method) and applied the hypergeometric test to identify enriched GO terms.

### Western Blot Analysis

Femoral nerve branches were rinsed in PBS buffer and lysed on ice in RIPA buffer containing a protease inhibitor cocktail (Pulilai), and the resulting tissue lysates were mixed with sample buffer and boiled at 95°C for 5 min. Equal amounts of protein from each sample were subjected to 10–15% sodium dodecyl sulfate-polyacrylamide gel electrophoresis and transferred to polyvinylidene fluoride membranes (Pulilai). The membranes were blocked in 5% nonfat dry milk at 4°C for 1 h and incubated with rabbit anti-MCP1 (1:1,000, ab25124, Abcam, Cambridge, MA, USA), rabbit anti-Inhibin beta A (1:1,000, ab56057, Abcam, Cambridge, MA, USA) and rabbit anti-HGF (1:1,000, ab83760, Abcam, Cambridge, MA, USA) primary antibody at 4°C overnight, followed by the appropriate secondary antibody, goat-anti-rabbit-HRP (1:3,000, GB23303, EPSILON), at room temperature for 1 h. The membranes were developed using an enhanced chemiluminescence substrate (Thermo Fisher Scientific, Waltham, MA, USA). Measurement of the protein band intensities was conducted using alphaEaseFC and Photoshop software.

### Histological Evaluation

#### Histological and Acetylcholinesterase (AChE) Staining

Transverse sections of the femoral nerve and its branches were cut to a 7 μm thickness on a cryostat microtome. The sections were fixed in acetone for 10 min and washed in PBS at room temperature to remove the OCT medium. Subsequently, a commercial hematoxylin and eosin (H&E) staining kit (G1120, Beijing Solarbio Science and Technology Company Limited, Beijing, China), a commercial Sirius Red staining kit (G1470, Beijing Solarbio Science and Technology Company Limited) and a commercial acetylcholinesterase (AChE) staining kit (G2110, Beijing Solarbio Science and Technology Company Limited) was used to stain the sections. All images were captured using a microscope equipped with a DP71 camera (BX51, Olympus, Tokyo, Japan). The above experiments were repeated three times independently and performed by three pathology staff under double-blind conditions.

#### Immunofluorescence Staining

The sections were blocked at room temperature with 10% goat serum in PBS for 1 h after washing three times for 5 min each in PBS. The primary mouse anti-laminin antibody (1:200, L8271, Sigma–Aldrich, St. Louis, MO, USA) was applied, and the sections were incubated in a humidified chamber overnight at 4°C. The next morning, the excess primary antibody was rinsed off with PBS. The sections were incubated with a goat anti-mouse IgG H&L secondary antibody (Alexa Fluor 594, 1:200, ab150116, Abcam, Cambridge, MA, USA) in PBS in the dark at room temperature for 2 h. After washing three times with PBS, the nuclei were counterstained with DAPI. Similar to the above immunofluorescence protocol, the primary rabbit anti-MCP1 antibody (1:100, ab25124, Abcam, Cambridge, MA, USA), rabbit anti-Inhibin beta A antibody (1:100, ab56057, Abcam, Cambridge, MA, USA) and rabbit anti-HGF antibody (1:100, ab83760, Abcam, Cambridge, MA, USA) were used as a marker for C-C motif chemokine 2 (encoded by the Ccl2 gene), Inhibin beta A chain (encoded by the Inhba gene) and Hepatocyte growth factor (encoded by the Hgf gene), respectively. Meanwhile, the goat anti-rabbit IgG H&L (Alexa Fluor 594, 1:200, ab150080, Abcam, Cambridge, MA, USA) was the secondary antibody. A microscope equipped with a DP71 camera was used to capture the immunofluorescence images. The above experiments were repeated three times independently and performed by three pathology staff under double-blind conditions.

### Ultrathin Sectioning

The nerve segments of the cutaneous and muscular branches were dehydrated through an ethanol series and embedded randomly in glycolmethacrylate mixed embedding medium to polymerize at gradually increasing temperature for 72 h. Transverse ultrathin sections of the nerve segments were cut randomly to a thickness of 70 nm using a diamond knife in an ultramicrotome (EM UC7, Leica Microsystems, Buffalo Springs, IL, USA) and collected in copper slot grids with pioloform/carbon support films. The ultrathin sections were counterstained with 3% lead citrate and uranyl acetate and observed by TEM (CM-120, Philips, Best, Netherlands). The ultrastructure of nerve branches were randomly photographed by TEM, then the pictures were numbered and submitted to the computer-generated random number table for selection (eight TEM images in each group). An ultra-thin slice of a nerve branch takes an average of eight photos. There are about 6–10 nerve fibers in each picture used to calculate the thickness of myelin sheath and G-ration (Figures 2A,B), and one nerve fiber in each picture used to calculate the thickness of the basement membrane (Figures 2C,D). After the stereology module was installed, the G-ratio and thickness of myelin sheath and BM and all nerve fibers in each photograph were calculated by the software of Image-Pro Plus 6.0. It is worth noting that G-ratio is obtained by calculating the ratio of axon diameter to nerve fiber diameter (axon and its outer myelin sheath). The above photographs were taken, selected and analyzed by three researchers in related fields under double-blind conditions.

**Figure 1:**
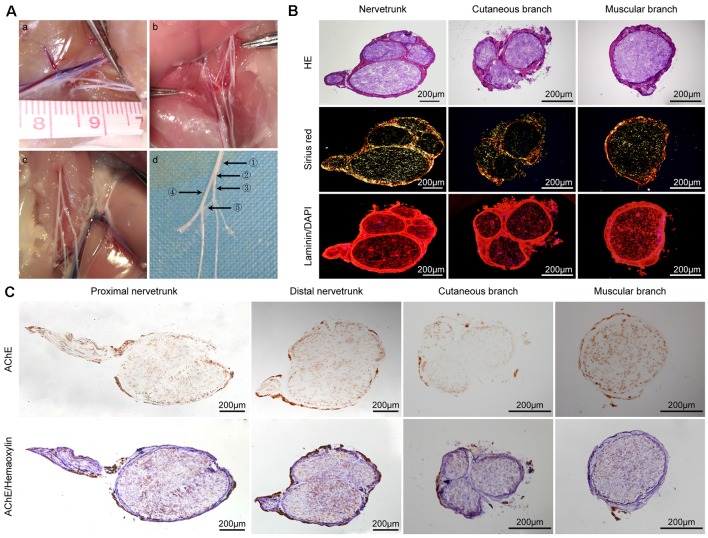
General observations and histological evaluation of the femoral nerve of rats. **(A)** General view of variant femoral nerves of rats. The different types of variant femoral nerves (a–c) and a magnified anatomical drawing (d) of graph (c). ①: proximal nerve trunk ②: distal nerve trunk ③: cutaneous branch ④: muscular branch ⑤: variant branch **(B)** Hematoxylin and eosin (H&E), Sirius Red, and laminin (red)/DAPI (blue) staining of the nerve trunk, cutaneous branch, and muscular branch of a normal rat femoral nerve, respectively. **(C)** Acetylcholinesterase (AChE) staining of the proximal nerve trunk, distal nerve trunk, cutaneous branch, and muscular branch of a rat normal femoral nerve; upper row is AChE staining only and the lower row is AChE and hematoxylin staining.

**Figure 2:**
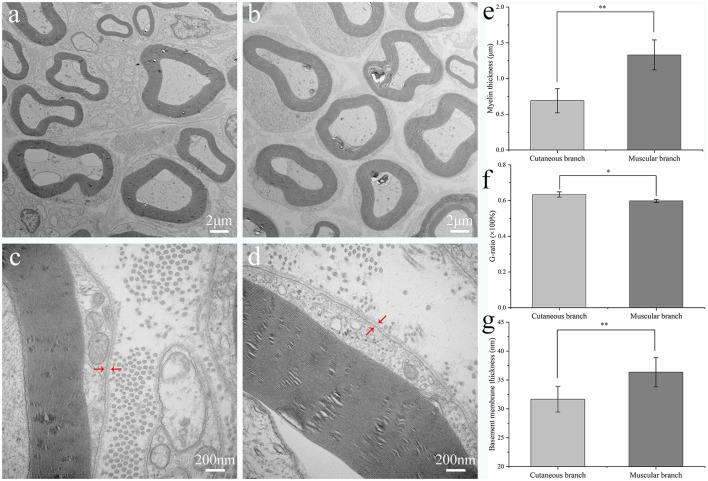
Ultrastructural evaluation of the rat femoral branches. Transmission electron microscopy of transverse ultrathin sections of the cutaneous branch **(A,C)** and muscular branch **(B,D)** of the rat normal femoral nerve. Arrowheads in **(C,D)** indicate the basement membranes of the femoral nerve branches. Mean thickness of the myelin sheath **(E)**, the value of G-ratio **(F)** and basement membrane **(G)** of the cutaneous and muscular branches of the rat normal femoral nerve. Wilcoxon rank-sum test was used in **(E)**, and independent-samples *t*-test were used in **(F)** and **(G)**, respectively. **p < 0.01, *p < 0.05.

### Statistical Analysis

Data are expressed as the mean ± SEM from at least three independent experiments. The G-ratio and thickness of myelin sheath and BM were analyzed by the image analysis software of Image-Pro Plus 6.0. For measurement data with equal variances, analysis of independent-samples *t*-test was used to analyze the differences between groups. For data with unequal variances, the Wilcoxon rank-sum test was used. The data were processed with SPSS 22.0 software (SPSS Inc., Chicago, IL, USA) and visualized using GraphPad Prism 6.0 (GraphPad Software Inc., La Jolla, CA, USA). A *P*-values < 0.05 were considered significant.

## Results

### Histology Evaluation

The femoral nerves of humans and rats are Y-shaped. However, during surgery, we found several types of variant femoral nerves in the rats (Figures 1Aa–c). Figure 1Ad is a magnified anatomical drawing of Figure 1Ac that shows a variant branch between the cutaneous and muscular branches. H&E staining revealed that several nerve fasciculi in the distal nerve trunk of the rat femoral nerve and the cutaneous branch had three nerve fasciculi. Sirius Red staining showed that the epineurium of the nerve trunk, cutaneous branch, and muscular branch mainly contained orange-yellow collagen I and the nerve fasciculi mainly contained green collagen III. The immunofluorescence analysis shows that the femoral nerve and its branch have a similar basement membrane (BM) tube (Figure 1B). AChE staining showed that both positive and negative sites were present on the femoral nerve trunk and there was a clear boundary in the distal nerve trunk, but an unclear boundary in the proximal nerve trunk. Interestingly, we also found a clear boundary in AChE staining of another proximal femoral trunk (Supplementary Figure S1). A large proportion of negative staining sites were detected on the cutaneous branch of the femoral nerve and only a few positive sites were found in the area near the epineurium. A large proportion of positive staining sites were detected on the femoral nerve muscular branches, whereas negative staining sites were unevenly distributed (Figure 1C).

### Ultrastructural Analysis

The myelin sheath and BM of the cutaneous and muscular branches were observed by TEM and analyzed statistically (Figure 2). There were eight TEM images selected to analyze the thickness of myelin sheath, BM and G-ratio from the group of the cutaneous branch (CB) and muscular branch (MB), respectively. The myelin sheath thickness of the medullated fibers of the muscular branch of the femoral nerve was greater than that of the cutaneous branch of the femoral nerve (1.33 ± 0.21 vs. 0.69 ± 0.17 μm, respectively; *p* < 0.01). The thickness of the BM of the muscular branch of the femoral nerve was greater than that of the cutaneous branch (36.36 ± 2.54 vs. 31.68 ± 2.23 nm, respectively; *p* < 0.01). However, the cutaneous branch has a larger value of G-ratio than muscular branch (0.6342 ± 0.0149 vs. 0.5982 ± 0.0081, respectively; *p* < 0.05).

### Identification of Proteins and Bioinformatics Analysis

#### Identification of Proteins

**Figure 3:**
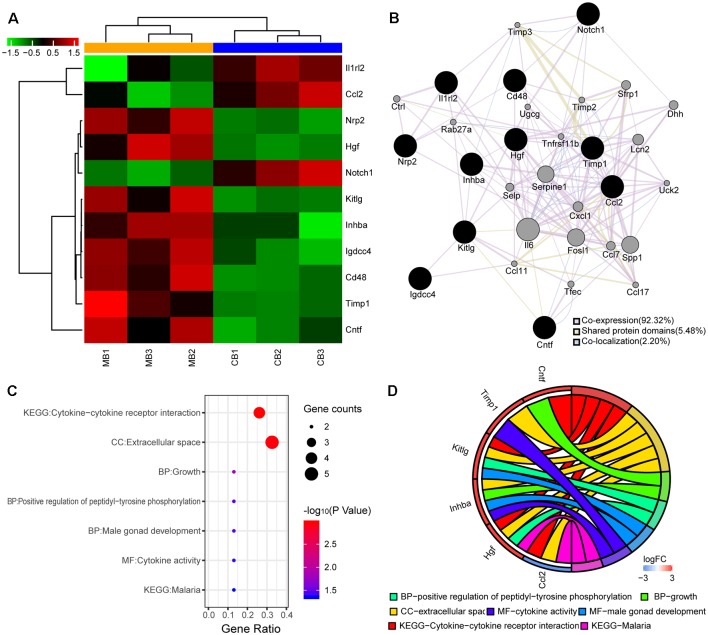
Differentially expressed proteins and bioinformatics analyses. **(A)** Heatmap shows up- and downregulated proteins. The horizontal axis indicates the protein name. Red and green represent the up- and downregulated proteins, respectively. The colors of the nodes are illustrated from red to green (black in the middle) in descending order of log2 (fold-change). **(B)** Protein-protein interaction (PPI) network of the differentially expressed proteins. The sizes of the spots in the network represent the weights of the interaction. The black spots represent the differentially expressed proteins determined experimentally and the gray spots represent the proteins predicted by GeneMANIA. **(C)** Enrichment analysis of 11 genes from the PPI network is shown in the bubble plot. The colors of the nodes are illustrated from red to blue in descending order of –log10 (*P*-value). The sizes of the nodes are illustrated from small to large in ascending order of gene counts. The horizontal axis represents the KEGG pathway and GO terms and the vertical axis represents the gene ratio. **(D)** The relationships among the top enriched KEGG pathway, GO terms, and differentially expressed genes in the PPI network. The relationships are represented by a chord plot in which genes are ordered according to their logFC values.

We examined the expression patterns of 67 proteins in the cutaneous and muscular branches using the Rat Cytokine Array 67. Proteins with a fold-change in expression >2 or < −2 and an adjusted *p* < 0.05 were defined as differentially expressed. A total of 11 proteins differentially expressed between the cutaneous and muscular branches were identified, of which three proteins were upregulated and eight were downregulated in the cutaneous branch compared with the muscular branch (Figure 3A).

#### PPI Network

A total of 11 proteins were differentially expressed between the cutaneous and muscular branches, suggesting an important difference between them. Consequently, a PPI network was constructed by uploading the up- and downregulated proteins into GeneMANIA. Co-expression characteristics, shared protein domains, and co-localization was observed in 92.32, 5.48, and 2.20% of the 11 differentially expressed proteins and their interacting proteins, respectively. The predictions, gene interactions, and pathways of all of the proteins are shown in Figure 3B.

#### GO and KEGG Pathway Analyses

A functional analysis was performed using DAVID to further explore the difference in the acquired proteins from the PPI networks between the cutaneous and muscular branches. As shown in Figure 3C, the results were divided into the GO and KEGG pathway analyses. In particular, the proteins classified in the BP category mainly consisted of three terms: positive regulation of peptidyl-tyrosine phosphorylation, male gonad development, and growth. The MF and CC categories mainly consisted of cytokine activities and extracellular space, respectively. The KEGG pathways of the targets were mainly related to cytokine–cytokine receptor interactions and malaria.

To visualize smaller subsets of the high-dimensional data, differentially expressed genes from the cutaneous and muscular branches were selected from the KEGG pathway and GO terms and presented in a chord plot using the GOplot package in R (Figure 3D).

### Western Blot and Immunofluorescence Analysis

**Figure 4:**
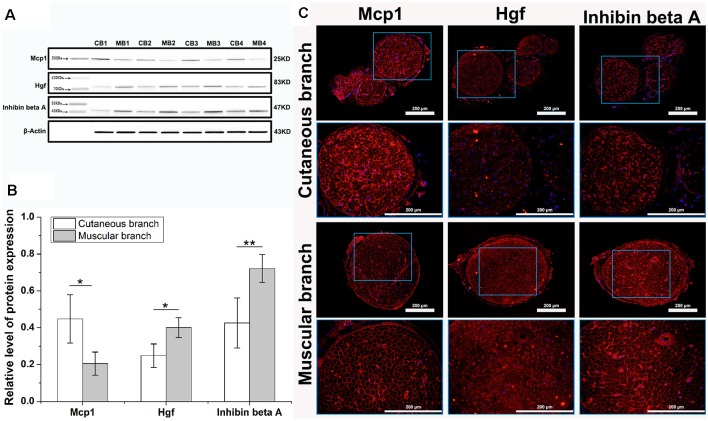
Western blot and immunofluorescence analysis of key differentially expressed proteins. **(A)** The western blot shows three specific protein bands, β-Actin served as an internal control. The Mcp1 protein was upregulated in cutaneous branch (CB), Hgf and Inhibin beta A proteins were upregulated in the muscular branch (MB). **(B)** The relative level of three key proteins expression, data are shown as means ± SD from four independent experiments, statistically analyzed by independent sample *t*-test. *p < 0.05; **p < 0.01. **(C)** The immunofluorescence image of three key differentially expressed proteins, each key protein (red) and nucleus (blue) are shown in the cutaneous branch and muscular branch of the femoral nerve. The interest area in the blue frame is enlarged in the following image.

To validate the analysis results of protein microarray, several key proteins were chosen for Western blot and immunofluorescence analysis. As we can see in the Figures 4A,B, the protein of Mcp1 (encoded by the Ccl2 gene), was upregulated in the cutaneous branch of femoral nerve, while the protein of Hgf and Inhibin beta A were upregulated in the muscular branch of the femoral nerve. Meanwhile, we have located the expression of key proteins using immunofluorescence. The Mcp1 protein is mainly expressed at the BM of nerve, the Hgf protein is mainly expressed at the myelin sheath of nerve and the Inhibin beta A protein is mainly expressed at the BM and myelin sheath of the nerve (Figure 4C).

## Discussion

We removed the rare variant femoral nerves of rats to ensure consistency and reliability of experiments (Figure 1Ac) and the two remaining variant nerves were found in our previous study (Figures 1Aa,b). According to the H&E and AChE staining results of the proximal nerve trunk, there were two nerve fasciculi, and the positive and negative AChE sites had an unclear boundary, indicating that the sensory and motor nerve fibers were mixed in the proximal nerve trunk of the femoral nerves of the rats. However, five nerve fasciculi were detected in the distal nerve trunk, and the sensory and motor nerve fibers had a relatively clear boundary, which indicates that the sensory and motor nerve fibers possess a complex journey. A previous study reported that the 3D high-resolution topography of the peripheral nerve fasciculus of humans can be reconstructed using an iodine and freeze-drying micro-computed tomography (microCT) method, which facilitates the acquisition of precise 3D topography of peripheral nerve fasciculi (Yan et al., 2017). The 3D internal microstructure of peripheral nerves at a static histological level has also been described (Delgado-Martínez et al., 2016). It will be useful to accurately visualize nerve injury and the repair necessary to achieve personalized treatment by combining the histological evaluation and the enhanced microCT imaging method.

As we all know, in previous studies, it is generally believed that the muscular branch of the femoral nerve is pure motor nerve fiber, and the cutaneous branch is pure sensory nerve fiber (Madison et al., 2007). Therefore, the femoral nerve plays an important role in the study of nerve regeneration (Irintchev et al., 2011). Interestingly, we found that the cutaneous branch of the femoral nerve is a mixed nerve mainly composed of sensory nerve fibers, and the muscular branch of the femoral nerve is also a mixed nerve mainly composed of motor nerve fibers. Meanwhile, it may have a functional significance that positive sites of AChE staining in cutaneous branches of the femoral nerve may be sympathetic postganglionic fibers distributed in sweat glands and erector pili muscles of the skin, and the negative part of AChE staining in muscular branches of the femoral nerve may be sensory nerve fibers from muscles, periosteum, and joints. Robinson and Madison (2006) once used the femoral nerve model to study the phenomenon of Chemotactic regeneration. However, after that, the related study found that the cutaneous branch of the femoral nerve is a pure sensory nerve, but the muscular branch of the femoral nerve is a mixed nerve, in which the number of sensory nerve fibers is more than that of motor nerve fibers (Peng et al., 2013). At the same time, through the resection of the dorsal root or ventral root of the spinal nerve, the animal model of pure sensory/motor nerve fibers can be established. In general, the femoral nerve model is the most suitable model to study Chemotactic regeneration. But if we want to obtain the animal model of pure motor/sensory nerve fibers, human intervention is necessary. The sciatic nerve model is usually used to evaluate the regeneration effect after nerve injury. Fu et al. (2020) found that axon regeneration and motor functional recovery were improved by electrical muscle stimulation with a rat sciatic nerve injury model. Otake et al. (2020) bridged sciatic nerve defect with OCT in rats and found that OCT can guide the growth of myelin sheath from the nerve stumps for the recovery of sensory function in the PNS. Compared with the sciatic nerve, the femoral nerve has two branches, the muscular branch and the cutaneous branch, which are often chosen in the study of chemotaxis regeneration. Therefore, for the rigor of the experiment, we chose the femoral nerve instead of the sciatic nerve in this study.

The myelin sheath is a membrane encapsulated outside the axons of nerve cells that insulates nerve signal propagation. Rapid nerve conduction requires the coating of axons to be a tightly packed multilayered myelin membrane. Myelin sheaths are critical for the physiological function of peripheral nerves, and enhancing remyelination improves functional recovery (Stassart et al., 2013). Tong et al. (2015) found that remyelination is diverse among myelinated motor and sensory nerve fibers. In our study, the mean thickness of the myelin sheath of the medullated fibers in the muscular branch of the femoral nerve was greater than that of the cutaneous branch, indicating that there should be a different standard of the thickness of the myelin sheath to evaluate remyelination of sensory and motor nerve fibers. Also, the variance of measurement data of the myelin thickness of the muscular branch is unequal (Figure 2C), there are two discrete value, which indicates that the muscular branch mainly contains motor nerve fibers, but a few sensory nerve fibers.

Nerve fibers with different diameters often have different conduction speeds. Generally speaking, the larger the diameter of a nerve fiber, the faster it will conduct. The conduction velocity of myelinated nerve fibers is proportional to the diameter. Therefore, the diameter distribution of myelinated axons can reflect the quality of regenerated neural tissue to a certain extent. The diameter of myelinated nerve fibers refers to the total diameter including axons and myelin. The ratio of the axon diameter to the total diameter (G-ratio) is closely related to the conduction velocity, and the most appropriate ratio is about 0.6 (Rushton, 1951). G-ratio is an index reflecting the maturity of regenerated myelinated nerve fibers. In our study, although the thickness of BM and myelin sheath of the cutaneous branch is smaller, it has a large value of G-ratio (0.6296 ± 0.008912 vs. 0.5869 ± 0.01608, respectively; *p* < 0.05). The value of the G-ratio of both branches is around 0.6, which is the most appropriate ratio. However, whether the statistical difference between the two branches has biological significance needs further experiments to verify. Ugrenović et al. (2016) reported that the optimal g-ratio of the sciatic nerve declines significantly with human age. de Campos et al. (2014) found that the g-ratio of the human recurrent laryngeal nerve between men and women is different, the parameters of the recurrent laryngeal nerve of the men are significantly larger than women, which may be related to physiological differences (de Campos et al., 2014).

The main components of the neural BM are members of the collagen family of proteins, the laminins and entactin (Yurchenco and Patton, 2009), which have neurite-promoting activity *in vitro* (Martini, 1994). The BM plays an important role in orderly reconstruction and the maintenance of tissue structure after injury, serving as a scaffold for cellular migration, arrangement, or attachment during Wallerian degeneration of the nerve (Giannini and Dyck, 1990). ANGs show reliable performance in the process of peripheral nerve regeneration following injury, in which the BM is the main structure with a well preserved 3D extracellular matrix structure (Hudson et al., 2004). In our study, the thickness of the BM of the muscular branches of the femoral nerve was greater than that of the cutaneous branch. 3D printing technology, also known as additive manufacturing, has made remarkable achievements in many fields. In recent years, with the rapid development of science and technology, nerve conduits prepared by 3D printing technology have also made certain progress in repairing peripheral nerve injuries. Vijayavenkataraman et al. (2019) fabricated 3D porous NGCs using a biodegradable and conductive block copolymer of PPy and a novel electrohydrodynamic jet 3D printing process, which can support higher growth of neural cells and a stronger maturation of hESC-NCSCs to peripheral neuronal cells. Tao et al. (2019) manufactured a functional nanoparticle-enhanced nerve conduit for promoting the regeneration of peripheral nerves, which consists of gelatin-methacryloyl (GelMA) hydrogels with drug-loaded poly (ethylene glycol)-poly (3-caprolactone; MPEG-PCL) nanoparticles dispersed in the hydrogel matrix and rapidly fabricated by a continuous 3D printing. It is unknown whether a better result would be obtained by bridging the nerve gap with a more suitable ANG and tissue engineered nerve grafts in which the BM is more similar in components and thickness to the injured nerve; therefore, related animal studies are ongoing.

We further evaluated the differentially expressed proteins between the cutaneous and muscular branches of the femoral nerve in rats. The results of the GO analysis indicated that five genes were enriched in the extracellular space term of the CC category (Figure 3D); expression of the *Ccl2* gene was upregulated in the cutaneous branch of the femoral nerve, and the four remaining genes (*Hgf*, *Inhba*, *Kitlg*, and *Timp2*) were upregulated in the muscular branch. These findings verified the ultrastructure of the femoral nerve branches. For example, a cell-cycle arrest is required for neuronal differentiation (Zhu and Skoultchi, 2001), and inhibited proliferation is correlated with the appearance of neurite outgrowth (Song et al., 2005). Leonor et al. reported that nerve growth factor acts synergistically with tissue inhibitor of metalloproteinase (TIMP)-2 to promote PC12 cell neurite outgrowth and partially rescued the reduced neurite length of TIMP-2^−/−^ cortical neurons *in vitro* (Pérez-Martínez and Jaworski, 2005). The key proteins, such as Mcp1, Hgf, and Inhibin beta A, are the parts of Extracellular space in CC, which were validated by Protein expression localization in immunofluorescence. At the same time, Quantitative key protein expression is consistent with the results of ultrastructural analysis.

Notably, this study is based only on normal femoral nerves, and variant femoral nerves of rats were excluded. Therefore, further study on variant femoral nerves should be conducted for a more comprehensive understanding. As we all know, with the development of 3D printing technology, tissue-engineered nerve grafts play an increasingly important role in the treatment of nerve defects. However, whether there are differences in the basement membrane of different types of nerves is unknown. Our study provides a new perspective for the development of ANGs and tissue-engineered nerve grafts with 3D printing technology using as a bridge for nerve gap in the treatment of nerve defects.

## Conclusion

In conclusion, there was an inherent difference in protein expression and ultrastructure between the cutaneous and muscular branches of the femoral nerve in rats, which will be important to consider in further studies on the treatment of peripheral nerve injuries.

## Data Availability Statement

The datasets generated for this study are available on request to the corresponding author.

## Ethics Statement

The animal study was reviewed and approved by The Ethics Committee of the Chinese People’s Liberation Army (PLA) General Hospital, China.

## Author Contributions

SW, QH, and XC: study design and article drafting. JM, XL, WX, and JP: experimental guidance and manuscript revision. XS and GH: data collection and analysis. XM and YW: study methods selection and technical support. All authors read and approved the final manuscript.

## Conflict of Interest

The authors declare that the research was conducted in the absence of any commercial or financial relationships that could be construed as a potential conflict of interest.

## Footnotes

^1^http://www.cytoscape.org/

## References

[B1] Abdullah M. O’Daly A. Vyas A. Rohde C. Brushart T. M. (2013). Adult motor axons preferentially reinnervate predegenerated muscle nerve. Exp. Neurol. 249, 1–7. 10.1016/j.expneurol.2013.07.019 23933577 PMC3818708

[B2] Cai B. B. Francis J. Brin M. F. Broide R. S. (2017). Botulinum neurotoxin type A-cleaved SNAP25 is confined to primary motor neurons and localized on the plasma membrane following intramuscular toxin injection. Neuroscience 352, 155–169. 10.1016/j.neuroscience.2017.03.049 28389376

[B3] Chen Z. L. Yu W. M. Strickland S. (2007). Peripheral regeneration. Annu. Rev. Neurosci. 30, 209–233. 10.1146/annurev.neuro.30.051606.094337 17341159

[B4] Cobianchi S. de Cruz J. Navarro X. (2014). Assessment of sensory thresholds and nociceptive fiber growth after sciatic nerve injury reveals the differential contribution of collateral reinnervation and nerve regeneration to neuropathic pain. Exp. Neurol. 255, 1–11. 10.1016/j.expneurol.2014.02.008 24552688

[B5] Cui X. Breitenkamp K. Finn M. G. Lotz M. D’Lima D. D. (2012). Direct human cartilage repair using three-dimensional bioprinting technology. Tissue Eng. Part A 18, 1304–1312. 10.1089/ten.tea.2011.0543 22394017 PMC3360507

[B6] de Campos D. Heck L. Jotz G. P. Xavier L. L. (2014). Degree of myelination (g-ratio) of the human recurrent laryngeal nerve. Eur. Arch. Otorhinolaryngol. 271, 1277–1281. 10.1007/s00405-013-2690-y 24061571

[B7] Delgado-Martínez I. Badia J. Pascual-Font A. Rodríguez-Baeza A. Navarro X. (2016). Fascicular topography of the human median nerve for neuroprosthetic surgery. Front. Neurosci. 10:286. 10.3389/fnins.2016.00286 27445660 PMC4929846

[B8] Fu T. Jiang L. Peng Y. Li Z. Liu S. Lu J.. (2020). Electrical muscle stimulation accelerates functional recovery after nerve injury. Neuroscience 426, 179–188. 10.1016/j.neuroscience.2019.10.052 31783103

[B9] Giannini C. Dyck P. J. (1990). The fate of Schwann cell basement membranes in permanently transected nerves. J. Neuropathol. Exp. Neurol. 49, 550–563. 10.1097/00005072-199011000-00002 2230836

[B10] He H. Y. Zhang J. Y. Mi X. Hu Y. Gu X. Y. (2015). Rapid prototyping for tissue-engineered bone scaffold by 3D printing and biocompatibility study. Int. J. Clin. Exp. Med. 8, 11777–11785. 26380018 PMC4565401

[B11] Huang da W. Sherman B. T. Lempicki R. A. (2009). Systematic and integrative analysis of large gene lists using DAVID bioinformatics resources. Nat. Protoc. 4, 44–57. 10.1038/nprot.2008.211 19131956

[B12] Hudson T. W. Zawko S. Deister C. Lundy S. Hu C. Y. Lee K.. (2004). Optimized acellular nerve graft is immunologically tolerated and supports regeneration. Tissue Eng. 10, 1641–1651. 10.1089/ten.2004.10.1641 15684673

[B14] Irintchev A. Wu M. M. Lee H. J. Zhu H. Feng Y. P. Liu Y. S.. (2011). Glycomimetic improves recovery after femoral injury in a non-human primate. J. Neurotrauma 28, 1295–1306. 10.1089/neu.2011.1775 21463132

[B15] Karabekmez F. E. Duymaz A. Moran S. L. (2009). Early clinical outcomes with the use of decellularized nerve allograft for repair of sensory defects within the hand. Hand 4, 245–249. 10.1007/s11552-009-9195-6 19412640 PMC2724628

[B16] Karegar M. Mohammadi R. (2015). Assessment of neuroregenerative effect of dihydrotestosterone, on peripheral nerve regeneration using allografts: a rat sciatic nerve model. Neurol. Res. 37, 908–915. 10.1179/1743132815y.0000000076 26187472

[B17] Langley J. N. Anderson H. K. (1904). The union of different kinds of nerve fibres. J. Physiol. 31, 365–391. 10.1113/jphysiol.1904.sp001042 16992733 PMC1465596

[B18] Lee B. Shafiq M. Jung Y. Park J. C. Kim S. H. (2016). Characterization and preparation of bio-tubular scaffolds for fabricating artificial vascular grafts by combining electrospinning and a co-culture system. Macromol. Res. 24, 131–142. 10.1007/s13233-016-4017-5 25557615

[B19] Madison R. D. Robinson G. A. Chadaram S. R. (2007). The specificity of motor neurone regeneration. Acta Physiol. 189, 201–206. 10.1111/j.1748-1716.2006.01657.x 17250570

[B20] Martini R. (1994). Expression and functional roles of neural cell surface molecules and extracellular matrix components during development and regeneration of peripheral nerves. J. Neurocytol. 23, 1–28. 10.1007/bf01189813 8176415

[B21] Otake K. Toriumi T. Ito T. Okuwa Y. Moriguchi K. Tanaka S.. (2020). Recovery of sensory function after the implantation of oriented-collagen tube into the resected rat sciatic nerve. Regen. Ther. 14, 48–58. 10.1016/j.reth.2019.12.004 31988995 PMC6965654

[B22] Peng J. P. Kou Y. H. Deng J. X. Zhang P. X. Yin X. F. Jiang B. G.. (2013). Generation and characterization of peripheral nerve animal model of pure motor/sensory nerve fibers. Beijing Da Xue Xue Bao Yi Xue Ban 45, 807–814. 24136284

[B23] Pérez-Martínez L. Jaworski D. M. (2005). Tissue inhibitor of metalloproteinase-2 promotes neuronal differentiation by acting as an anti-mitogenic signal. J. Neurosci. 25, 4917–4929. 10.1523/JNEUROSCI.5066-04.2005 15901773 PMC1282460

[B24] Robinson G. A. Madison R. D. (2006). Developmentally regulated changes in femoral nerve regeneration in the mouse and rat. Exp. Neurol. 197, 341–346. 10.1016/j.expneurol.2005.10.007 16300759

[B25] Rushton W. A. (1951). A theory of the effects of fibre size in medullated nerve. J. Physiol. 115, 101–122. 10.1113/jphysiol.1951.sp004655 14889433 PMC1392008

[B26] Sachanandani N. F. Pothula A. Tung T. H. (2014). Nerve gaps. Plast. Reconstr. Surg. 133, 313–319. 10.1097/01.prs.0000436856.55398.0f 24150118

[B27] Sameem M. Wood T. J. Bain J. R. (2011). A systematic review on the use of fibrin glue for peripheral nerve repair. Plast. Reconstr. Surg. 127, 2381–2390. 10.1097/prs.0b013e3182131cf5 21311390

[B28] Song J. H. Wang C. X. Song D. K. Wang P. Shuaib A. Hao C. (2005). Interferon γ induces neurite outgrowth by up-regulation of p35 neuron-specific cyclin-dependent kinase 5 activator *via* activation of ERK1/2 pathway. J. Biol. Chem. 280, 12896–12901. 10.1074/jbc.M412139200 15695523

[B29] Stassart R. M. Fledrich R. Velanac V. Brinkmann B. G. Schwab M. H. Meijer D.. (2013). A role for Schwann cell-derived neuregulin-1 in remyelination. Nat. Neurosci. 16, 48–76. 10.1038/nn.3281 23222914

[B30] Tao J. Zhang J. Du T. Xu X. Deng X. Chen S.. (2019). Rapid 3D printing of functional nanoparticle-enhanced conduits for effective nerve repair. Acta Biomater. 90, 49–59. 10.1016/j.actbio.2019.03.047 30930306

[B31] Tong L. L. Ding Y. Q. Jing H. B. Li X. Y. Qi J. G. (2015). Differential motor and sensory functional recovery in male but not female adult rats is associated with remyelination rather than axon regeneration after sciatic nerve crush. Neuroreport 26, 429–437. 10.1097/wnr.0000000000000366 25830493

[B32] Turkof E. Jurasch N. Knolle E. Schwendenwein I. Habib D. Unger E.. (2006). Motor evoked potentials enable differentiation between motor and sensory branches of peripheral nerves in animal experiments. J. Reconstr. Microsurg. 22, 525–532. 10.1055/s-2006-951318 17048135

[B33] Ugrenović S. Jovanović I. Vasović L. Kundalić B. Čukuranović R. Stefanović V. (2016). Morphometric analysis of the diameter and g-ratio of the myelinated nerve fibers of the human sciatic nerve during the aging process. Anat. Sci. Int. 91, 238–245. 10.1007/s12565-015-0287-9 25976073

[B34] Vijayavenkataraman S. Kannan S. Cao T. Fuh J. Y. H. Sriram G. Lu W. F. (2019). 3D-printed PCL/PPy conductive scaffolds as three-dimensional porous nerve guide conduits (ngcs) for peripheral nerve injury repair. Front. Bioeng. Biotechnol. 7:266. 10.3389/fbioe.2019.00266 31750293 PMC6843025

[B35] Wang D. Liu X. L. Zhu J. K. Hu J. Jiang L. Zhang Y.. (2010). Repairing large radial nerve defects by acellular nerve allografts seeded with autologous bone marrow stromal cells in a monkey model. J. Neurotrauma 27, 1935–1943. 10.1089/neu.2010.1352 20701436

[B36] Yan L. Guo Y. Qi J. Zhu Q. Gu L. Zheng C.. (2017). Iodine and freeze-drying enhanced high-resolution MicroCT imaging for reconstructing 3D intraneural topography of human peripheral nerve fascicles. J. Neurosci. Methods 287, 58–67. 10.1016/j.jneumeth.2017.06.009 28634148

[B37] Yurchenco P. D. Patton B. L. (2009). Developmental and pathogenic mechanisms of basement membrane assembly. Curr. Pharm. Des. 15, 1277–1294. 10.2174/138161209787846766 19355968 PMC2978668

[B38] Zhang Y. Luo H. Zhang Z. Lu Y. Huang X. Yang L.. (2010). A nerve graft constructed with xenogeneic acellular nerve matrix and autologous adipose-derived mesenchymal stem cells. Biomaterials 31, 5312–5324. 10.1016/j.biomaterials.2010.03.029 20381139

[B39] Zhu L. Skoultchi A. I. (2001). Coordinating cell proliferation and differentiation. Curr. Opin. Genet. Dev. 11, 91–97. 10.1016/s0959-437x(00)00162-3 11163157

[B40] Zuberi K. Franz M. Rodriguez H. Montojo J. Lopes C. T. Bader G. D.. (2013). GeneMANIA prediction server 2013 update. Nucleic Acids Res. 41, W115–W122. 10.1093/nar/gkt533 23794635 PMC3692113

