# Supplementary material for: Differences in the Structure and Protein Expression of Femoral Nerve Branches in Rats
Source: Front Neuroanat. 2020 Apr 8;14:16. doi: 10.3389/fnana.2020.00016 (PMC7156789; doi:10.3389/fnana.2020.00016)
Supplement: Supplementary file 1 [file Table_1.DOCX]

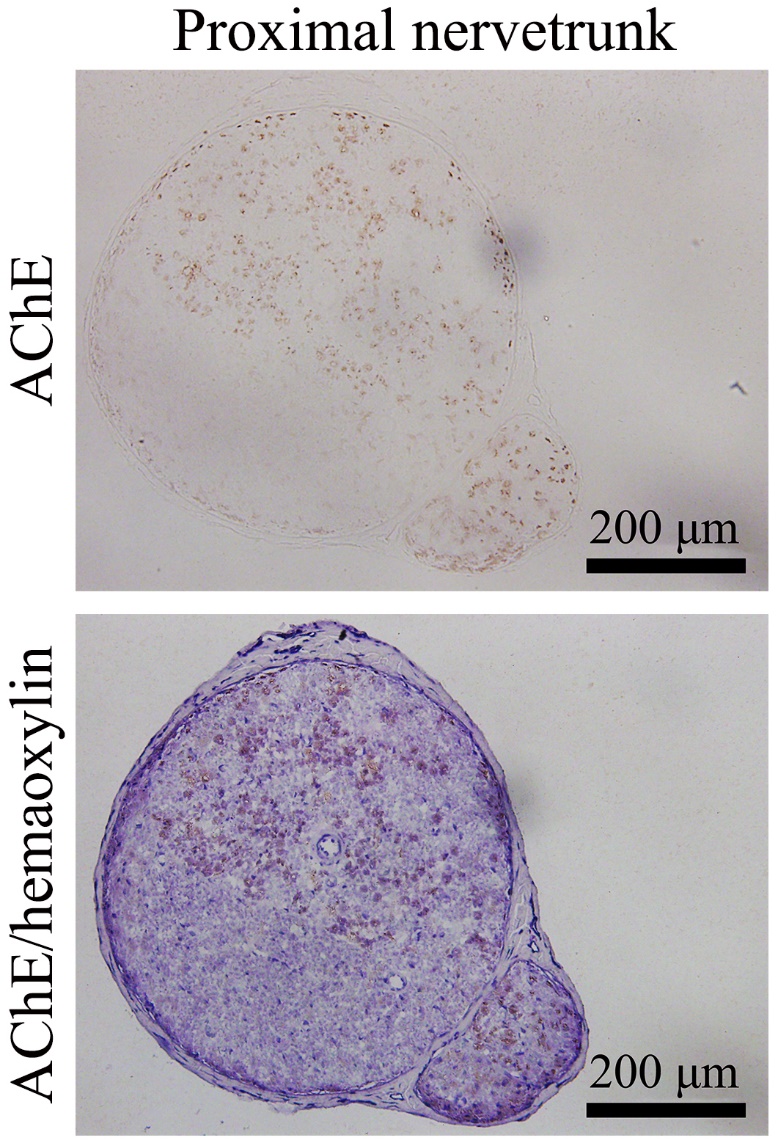


Supplementary Figure 1

**Supplementary Figure. 1.** Acetylcholinesterase (AChE) staining of the proximal nerve trunk of a rat normal femoral nerve; upper row is AChE staining only and the lower row is AChE and hemaoxylin staining.
